# Microbicidal activity of N-chlorotaurine in combination with hydrogen peroxide

**DOI:** 10.1186/s13568-017-0404-3

**Published:** 2017-05-23

**Authors:** Jasmin Mustedanagic, Valdecir Farias Ximenes, Markus Nagl

**Affiliations:** 1 Division of Hygiene and Medical Microbiology, Medical University of Innsbruck, Innsbruck, Austria; 2 Department of Chemistry, Faculty of Science, UNESP - São Paulo State University, Bauru, SP Brazil; 3 Division of Hygiene and Medical Microbiology, Medical University of Innsbruck, Schöpfstr. 41, 1st Floor, 6020 Innsbruck, Austria

**Keywords:** *N*-Chlorotaurine, Hydrogen peroxide, Singlet oxygen, Microbicidal, Antimicrobial agent, Oxidant

## Abstract

*N*-chlorotaurine (NCT) and hydrogen peroxide are powerful endogenous antiseptics. In vivo, the reaction between hydrogen peroxide and metal ions leads to the formation of free hydroxyl radicals, which have an increased bactericidal activity. This study examined whether there is an additive antimicrobial effect of NCT combined with hydrogen peroxide. Additionally, it was tested if the additive effect is based on the formation of free radicals. We found by luminometry that, in the presence of H_2_O_2_, NCT caused a slow and long-lasting production of singlet oxygen in contrast to HOCl, where this burst occurred instantaneously. Both NCT and hydrogen peroxide (1.0 and 0.1%) demonstrated bactericidal and fungicidal activity. At pH 7.1 and 37 °C, hydrogen peroxide (1%, 294 mM) showed a stronger bactericidal and particularly fungicidal activity than NCT (1%, 55 mM), whereas at pH 4.0 and also in the presence of 5.0% peptone NCT revealed a stronger bactericidal activity. A combination of NCT and hydrogen peroxide led to an increased bactericidal but no increased fungicidal activity compared to both substances alone. The additive effect against bacteria was not removed in the presence of the radical scavengers NaN_3_, DMSO, or peptone. As a conclusion, NCT and hydrogen peroxide used concurrently interact additive against a range of microorganisms. However, the results of this study suggest that the additive effect of NCT combined with hydrogen peroxide is rather not based on the formation of free radicals.

## Introduction

Human phagocytes are activated by invading microorganisms and produce several reactive oxygen species (ROS) by an enzymatic cascade called oxidative burst (Klebanoff 1968). Superoxide (O_2_.^−^) and hydrogen peroxide (H_2_O_2_) are products of NADPH oxidase and superoxide dismutase, respectively, and hypochlorous acid (HOCl) is formed from H_2_O_2_ and chloride by myeloperoxidase (Weiss et al. 1982; Zgliczynski et al. 1971). HOCl immediately reacts among others with amino groups to create chloramines (R-NHCl), also designated as long-lived oxidants because of their lower reactivity. *N*-chlorotaurine (Cl–HN–CH_2_–CH_2_–SO_3_^−^, NCT) is the most abundant representative of this class of compounds (Grisham et al. 1984). All these oxidants are thought to be involved in the killing of microorganisms during inflammation (Klebanoff et al. 2013). However, their spectrum of functions is broader, such as signal transduction by H_2_O_2_ (Kim et al. 2002; Mongkolsuk and Helmann 2002), or anti-inflammatory effects by NCT (Kim and Cha 2014; Marcinkiewicz and Kontny 2014). Moreover, chemically synthesized H_2_O_2_ and HOCl are in use as antiseptics in human medicine (Bruch 2007; Wilkins and Unverdorben 2013), and NCT is particularly suited for treatment of infections of sensitive body sites according to several studies (Gottardi and Nagl 2010; Nagl et al. 2003; Neher et al. 2004; Teuchner et al. 2005).

As an interesting aspect, the mentioned oxidants may also react among one another, whereby further microbicidal ROS are formed. The reaction between HOCl and H_2_O_2_ to form singlet oxygen (^1^O_2_) (Eq. 1) was discovered by Khan and Kasha in (1963), and it was studied in details due to its physiological and pathological relevance, including its involvement in signaling and microbicidal functions (Khan and Kasha 1963; Steinbeck et al. 1992; Stief and Fareed 2000).

1 $${\text{H}}_{2} {\text{O}}_{2} + {\text{OCl}}^{ -} \rightarrow {}^{1}{\text{O}}_{2} + {\text{Cl}}^{ - } + {\text{H}}_{2} {\text{O}_{2}}$$

The oxidation of H_2_O_2_ leading to ^1^O_2_ is not a property exclusive of HOCl (Miyamoto et al. 2014), but also shared by other halogenating species as hypobromous acid (HOBr) (Kanofsky et al. 1988), chloramines, including NCT (Stief 2003) and N-bromotaurine (De Carvalho et al. 2016). However, although the reactivity of NCT with H_2_O_2_ leading to ^1^O_2_ has been proposed (Stief et al. 2001; Stief 2003), as far as we know, it was never investigated in details. Here we identified an advantage in the use of NCT compared to HOCl regarding the production of ^1^O_2_.

The aim of this study was to characterize the reaction of NCT and hydrogen peroxide with production of singlet oxygen and to investigate a possible additive bactericidal or fungicidal effect of both compounds.

## Materials and methods

### Chemicals

Taurine, melatonin, hydrogen peroxide and deuterium oxide were purchased from Sigma-Aldrich Chemical Co. (St. Louis, MO, USA). Working solution of hypochlorous acid (HOCl) 100 mM was prepared by diluting a 5% commercial solution in water. The concentration of HOCl was determined spectrophotometrically after diluting the working solution in 0.01 M NaOH, pH 12 (λ_max_ = 292 nm, ε = 350/M cm). *N*-Chlorotaurine (NCT) (molecular weight 181.57 g/mol, lot 2015-02-05) was prepared as crystalline sodium salt in our laboratory (M. Nagl, Innsbruck) at pharmaceutical grade, as reported (Gottardi and Nagl 2002), stored at minus 20 °C, and freshly dissolved in sterile 0.1 mM sodium phosphate buffer at pH 7.1 to a concentration of 55 mM (1%), 5.5 mM (0.1%), 1 mM (0.018%) or 0.25 mM (0.0045%) for each experiment.

Hydrogen peroxide (H_2_O_2_) 100 mM was prepared by dilution of a 30% commercial solution (Merck, Darmstadt, Germany) in water. The concentration of H_2_O_2_ was determined spectrophotometrically by its absorbance (λ_max_ = 240 nm, ε = 43.6/M cm). The chemicals used for preparation of phosphate buffer and solutions were of analytical grade. Ultrapure Milli-Q water (Millipore, Belford, MA, USA) was used for the preparation of buffers and solutions. Bacto™ peptone from Becton–Dickinson and Company (NJ, USA) was dissolved in distilled water to a 10% stock solution and autoclaved. Sodium azide (NaN_3_) was dissolved in distilled water to a 0.65% (100 mM) stock, dimethyl sulfoxide (DMSO) to a 10% stock (1.28 M) in 0.1 M phosphate buffer. Catalase from *Micrococcus lysodeikticus* containing 65,000–150,000 U/ml was from Sigma-Aldrich (Germany). Chloramine T from Merck was dissolved in 0.1 M phosphate buffer to 0.005% (0.178 mM).

### Studies of the reaction between NCT or HOCl and H_2_O_2_

Consumption of NCT and HOCl were monitored by their absorbances at 252 and 290 nm, respectively, using a Perkin Elmer Lambda 25 UV–visible spectrophotometer (Shelton, CT, USA). The reaction mixtures were composed of 0.25 mM NCT or HOCl, and 0.5 mM H_2_O_2_ in 0.1 M phosphate buffer, pH 7.1 and 37 °C. The production of ^1^O_2_ was monitored by its dimol light emission or, indirectly, by the light emission generated by its reaction with melatonin using a plate luminometer (Centro Microplate Luminometer LB960, Berthold Technologies, Oak Ridge, TN, USA). The reaction mixtures were composed of 1.0 mM NCT or HOCl, 20 mM H_2_O_2_, in the presence or absence of 1 mM melatonin in 0.1 M phosphate buffer, pH 7.1 and 37 °C. The reactions were triggered by the addition of H_2_O_2_.

### Bacteria and fungi

Bacteria and yeasts deep frozen for storage were grown on Mueller–Hinton agar plates (Oxoid, Hampshire, UK) and subcultivated overnight in tryptic soy broth (Merck) at 37 °C. Subsequently, they were washed twice in 0.9% saline before use. Strains used were *Staphylococcus aureus* ATCC 25923 and 6538, *Pseudomonas aeruginosa* ATCC 27853, *Escherichia coli* ATCC 11229, and *Candida albicans* CBS 5982 (60% pseudohyphae and 40% blastoconidia). *Aspergillus fumigatus* ATCC 204305 was grown on Sabouraud agar (Becton & Dickinson, Heidelberg, Germany) for 72 h. Suspensions of conidia were gained by harvesting them from the agar plates with 5.0 ml of 0.9% saline plus 0.01% Tween 20, followed by 10-µm filtration (CellTrics; Partec GmbH, Görlitz, Germany) to gain a pure conidia suspension without hyphae and three washing steps in phosphate-buffered saline (Lackner et al. 2015).

### Time-kill assays (Lackner et al. 2015; Martini et al. 2012)

All experiments were done at 37 °C in a water bath. NCT (1.98 ml) was mixed with H_2_O_2_ (1.98 ml) in 0.1 M phosphate buffer (pH 7.1) or 0.1 M sodium acetate buffer (pH 4.0) to final concentrations of 1% (equals 55 mM NCT and 294 mM H_2_O_2_) or 0.1% each. In parallel, 3.96 ml of 1 or 0.1% NCT and 3.96 ml of 1 or 0.1% H_2_O_2_ were investigated in each experiment. A control in phosphate or acetate buffer without additives was done in parallel. In separate experiments, single radical scavengers were added to all test tubes. Thereby, respective volumes of the stock solutions were mixed immediately before the start of the test, for instance 1.98 ml of 2% NCT plus 2% H_2_O_2_ with 1.98 ml 10% peptone. Final concentrations were 5% peptone, 5% DMSO, or 10 mM sodium azide. To 3.96 ml of the test solutions, 40 µl of the respective suspension containing bacteria or fungi were added at time zero and vortexed. Final starting concentrations of microorganisms were 1.6–6.6 × 10^6^ colony forming units (cfu)/ml for *S. aureus* ATCC 25923, 1.1–2.3 × 10^7^ cfu/ml for *S. aureus* ATCC 6538, 1.1–3.1 × 10^7^ cfu/ml for *P. aeruginosa* and *E. coli*, 2.0–4.4 × 10^5^ cfu/ml for *C. albicans*, 4.4–1.0 × 10^6^ cfu/ml for *A. fumigatus*. Incubation times ranged between 1 and 240 min and are indicated in the figures. At the end of each incubation time, aliquots of 100 µl were diluted in 900 µl NCT-inactivating solution consisting of 890 µl of 3% sodium thiosulphate plus 10 µl (approximately 1000 U) catalase in distilled water. Aliquots of 50 µl of this solution were spread on Mueller–Hinton agar plates in duplicate using an automatic spiral plater (model WASP 2, Don Whitley, Shipley, United Kingdom). The detection limit was 100 cfu/ml, taking into account both plates and the previous 10-fold dilution in the inactivating solution. Plates were grown for 48 h (bacteria) to 72 h (fungi) at 37 °C, and the number of cfu was counted. Plates with no growth or only a low cfu count were grown for up to 5 days (bacteria, *Candida*) or ten days (*Aspergillus*). Controls, i.e. plain 0.1 M phosphate buffer with and without scavengers (peptone, DMSO, NaN_3_) were performed in parallel. Inactivation controls, where NCT and H_2_O_2_ were mixed with their inactivators thiosulphate plus catalase immediately before addition of pathogens at low cfu counts, showed full survival of bacteria and fungi. This proved rapid and sufficient inactivation.

### Time-kill assays with sequential treatment of NCT and H_2_O_2_

To investigate if there is an additive bactericidal effect after sequential incubation in both compounds, the pellet of washed bacteria (*S. aureus* ATCC 6538, *E. coli*, *P. aeruginosa*) was resuspended in the slower acting agent, 1% NCT, in 0.1 M phosphate buffer (pH 7.1) to 2–5 × 10^9^ cfu/ml at room temperature first. The incubation time was 1 min. Controls were treated with phosphate buffer without NCT. After that time, the cfu count is not influenced, but the surface of the bacteria becomes chlorinated (“chlorine cover”) (Gottardi and Nagl 2005). Then, the bacteria were centrifuged at 4000×*g* for 5 min, washed in 0.9% NaCl, centrifuged again, and resuspended in saline. Subsequently, 40 µl of the bacterial suspension was added to 3.96 ml 1% H_2_O_2_ (0.3% H_2_O_2_ for tests with *E. coli* and *P. aeruginosa*) in 0.1 M phosphate buffer (pH 7.1), and incubated at 37 °C (controls in buffer without H_2_O_2_). Quantitative cultures from aliquots after different incubation times were performed as described in the previous paragraph.

### Statistics

The data are presented as mean values and standard deviations (SD) of at least three independent experiments. Student’s unpaired *t* test in case of two groups or one-way analysis of variance (ANOVA) and Tukey’s multiple-comparison test in case of more than two groups were used to test for a difference between the test and control groups. A *P* value of <0.05 was considered significant for all tests. Calculations were done with the GraphPad Prism 6.01 software (GraphPad, Inc., La Jolla, CA, USA).

To gain an improved survey on the microbicidal activity of NCT against the different strains, the recently introduced Integral Method was used, which transforms the whole killing curve (log_10_ cfu/ml versus time) into one value of “bactericidal activity (BA)” (Gottardi et al. 2015). The higher the value, the stronger is the microbicidal activity. Moreover, the method allows an expanded statistical analysis with the tests mentioned above, particularly between killing curves with small differences.

## Results

### Reaction between NCT or HOCl and H_2_O_2_

**Fig. 1:**
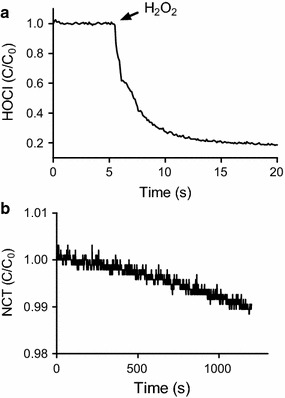
Reaction of HOCl (**a**) and NCT (**b**) with H_2_O_2_. The reactions were monitored by HOCl and NCT absorbance decay. One representative experiment of 3 independent ones with similar outcome is depicted

**Fig. 2:**
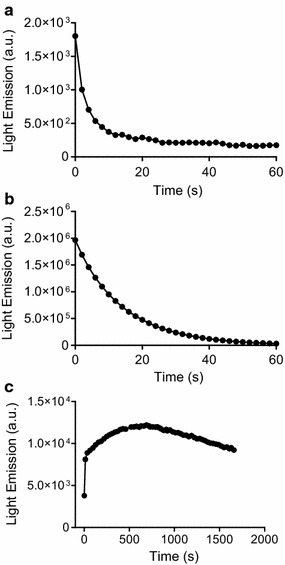
Light emission generated by the production of singlet oxygen. **a** HOCl and H_2_O_2_; **b** HOCl, H_2_O_2_ and melatonin; **c** NCT, H_2_O_2_ and melatonin. Reaction condition: HOCl and NCT 1 mM, melatonin 1 mM, H_2_O_2_ 1 mM in 0.1 M phosphate buffer, pH 7.1 and 37 °C. One representative experiment of 3 independent ones with similar outcome is depicted

**Fig. 3:**
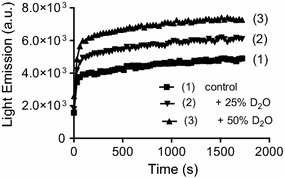
Effect of deuterium oxide in the light emission. Control reaction: NCT 1 mM, melatonin 1 mM, H_2_O_2_ 3 mM in 0.1 M glycine buffer pH 9.0 and 37 °C. One representative experiment of 3 independent ones with similar outcome is depicted

We found that, differently of HOCl, which produces an instantaneous burst of ^1^O_2_ in the presence of H_2_O_2_, NCT caused a slow and long-lasting production of this electronically excited form of molecular oxygen. Figure 1 shows the consumption of HOCl and NCT provoked by the addition of H_2_O_2_. While HOCl was almost completely depleted in less than 5 s, NCT lost less than 2% of its initial concentration in 20 min. The reactions were also monitored by the weak chemiluminescence generated by dimol emission of ^1^O_2_ (Lengfelder et al. 1983). Figure 2a shows that, although of low intensity and of a short period (less than 10 s), chemiluminescence was detected by the reaction between HOCl and H_2_O_2_. On the other hand, due to its lower reactivity, light emission was not detected using NCT. Hence, to improve the efficiency, we added melatonin, which promptly reacts with ^1^O_2_ leading to light emission through the formation of an unstable dioxetane intermediate (Lu et al. 2002; Matuszak et al. 2003). The result in Fig. 2b shows that the emission of light for the reaction between HOCl and H_2_O_2_ was increased about 3 orders of magnitude in the presence of melatonin, reinforcing the involvement of ^1^O_2_. Finally, Fig. 3 shows the kinetic profile of light emission when HOCl was substituted by NCT in the presence of melatonin and H_2_O_2_. Corroborating with the results obtained by monitoring the consumption of NCT, the production of ^1^O_2_ took place in more than 30 min, which is an evidence of the slow, but efficient reactivity of this chloramine with H_2_O_2_. Of note, according controls disclosed that light emission was not due to direct reaction of melatonin with H_2_O_2_ or with NCT (not showed). We also found that the reaction rate was still lower at alkaline pH, which is consistent with the lower reactivity of NCT in this condition. Additional evidence of the formation of ^1^O_2_ was obtained by addition of deuterated water in the reaction medium, which increase its lifetime (Kim et al. 2016) and, consequently, the efficiency of the reaction (Fig. 3).

### Microbicidal activity of NCT and H_2_O_2_

**Fig. 4:**
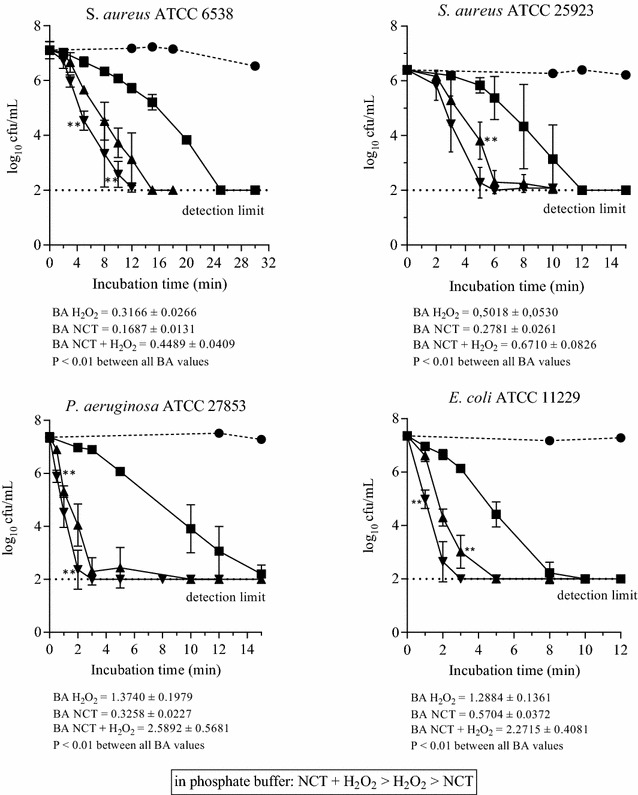
Bactericidal activity of 1% NCT (*filled square*), 1% H_2_O_2_ (*filled triangle*), and 1% NCT plus 1% H_2_O_2_ (*inverted triangle*) at pH 7.1 and 37 °C. Control in phosphate buffer without additives (*filled circle*). Mean values ± SD of three to four independent experiments. **P < 0.01 versus all other values. BA “bactericidal activity” [log_10_ cfu/min] as a quantitative measure for the strength of killing calculated by the integral method for the whole killing curve according to (Gottardi et al. 2015). The higher the value, the higher the microbicidal activity

**Fig. 5:**
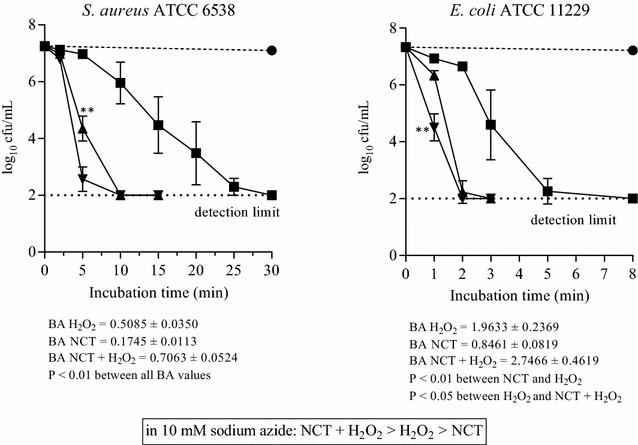
Bactericidal activity of 1% NCT (*filled square*), 1% H_2_O_2_ (*filled triangle*), and 1% NCT plus 1% H_2_O_2_ (*inverted triangle*) at pH 7.1 and 37 °C in the presence of 10 mM sodium azide (NaN_3_). Control in phosphate buffer plus 10 mM NaN_3_ (*filled circle*). Mean values ± SD of three (*E. coli*) to four (*S. aureus*) independent experiments. **P < 0.01 versus all other values. BA values calculated as in Fig. 4

**Fig. 6:**
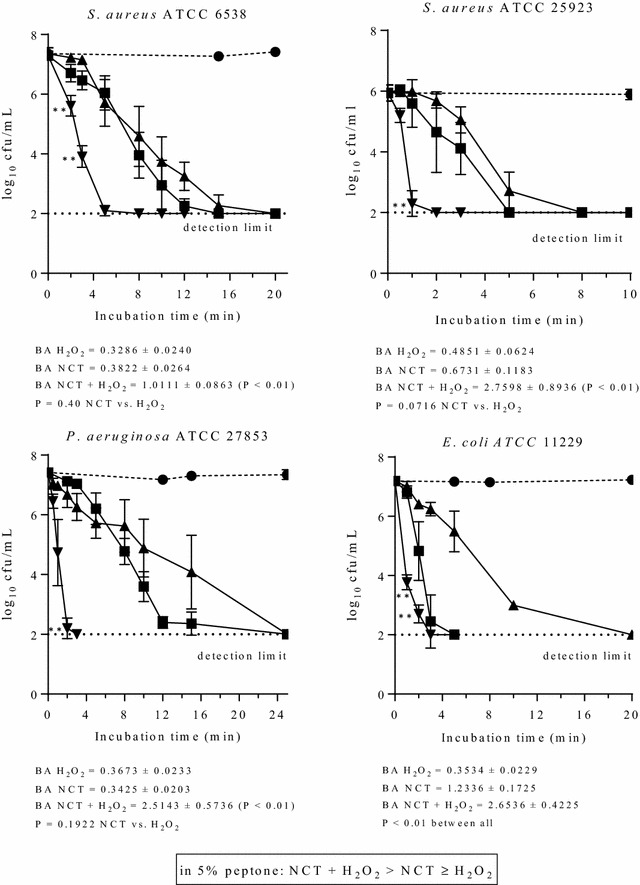
Bactericidal activity of 1% NCT (*filled square*), 1% H_2_O_2_ (*filled triangle*), and 1% NCT plus 1% H_2_O_2_ (*inverted triangle*) at pH 7.1 and 37 °C in the presence of 5% peptone. Control in phosphate buffer plus 5% peptone (*filled circle*). Mean values ± SD of three to four independent experiments. **P < 0.01 versus all other values. BA values calculated as in Fig. 4

Against all bacterial test strains, the combination of NCT and H_2_O_2_ showed a more rapid killing than the single compounds. In phosphate buffer at pH 7.1, H_2_O_2_ had a throughout stronger activity than NCT. This is illustrated in Fig. 4. To investigate the influence of singlet oxygen formed by NCT plus H_2_O_2_, sodium azide as a scavenger was added to the test solutions at a concentration of 10 mM, which is a threshold that just does not kill bacteria (Sabbahi et al. 2008). Surprisingly, the significantly more rapid killing by the combination was still present (Fig. 5). The same was true if 5% DMSO, a hydroxyl radical scavenger, was added (data not shown). Finally, we added 5% peptone as a general quencher of oxidants (Fig. 6). Of note, NCT plus H_2_O_2_ was still the highly significantly strongest bactericidal solution. In addition, the already known enhancing effect of organic matter on NCT was seen again (Gottardi et al. 2014), while H_2_O_2_ was weakened. Therefore, NCT turned out to kill rather more rapidly than H_2_O_2_ under these conditions.

A similar result was seen at pH 4 in acetate buffer where 0.1% NCT and 0.1% H_2_O_2_ were tested. Again, the combination showed the strongest effect followed by NCT and H_2_O_2_ (BA = 0.98 ± 0.15 log_10_ cfu/min for NCT plus H_2_O_2_, 0.60 ± 0.07 for NCT, and 0.25 ± 0.02 for H_2_O_2_).

**Fig. 7:**
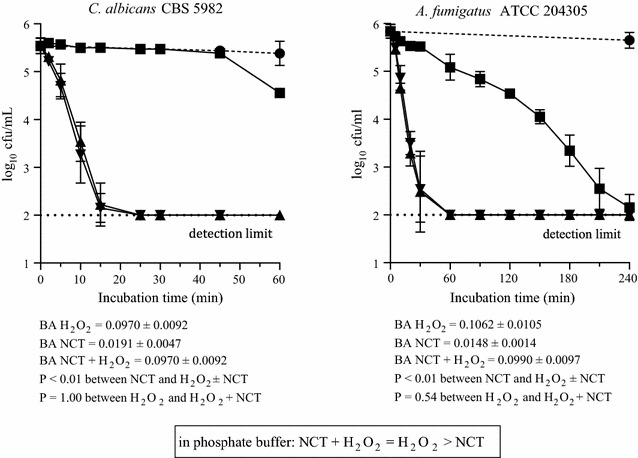
Fungicidal activity of 1% NCT (*filled square*), 1% H_2_O_2_ (*filled triangle*), and 1% NCT plus 1% H_2_O_2_ (*inverted triangle*) at pH 7.1 and 37 °C. Control in phosphate buffer without additives (*filled circle*). Mean values ± SD of three to four independent experiments. BA values calculated as in Fig. 4

Against *C. albicans* and *A. fumigatus* in phosphate buffer, no difference was found between NCT plus H_2_O_2_ and H_2_O_2_ alone (Fig. 7), while NCT killed fungi much slower than bacteria as expected from former work (Nagl et al. 2001).

In some experiments, 0.005% CAT was used instead of 1% NCT against *S. aureus* 6538. The BA value of 1% H_2_O_2_ was 0.28 ± 0.02, that of CAT 0.55 ± 0.05 and that of CAT plus 1% H_2_O_2_ 0.67 ± 0.07 log_10_ cfu/min. The difference between CAT and CAT plus H_2_O_2_ did not reach significance but a trend after 3 independent experiments (*P* = 0.0676).

### Time-kill assays with sequential treatment of NCT and H_2_O_2_

**Fig. 8:**
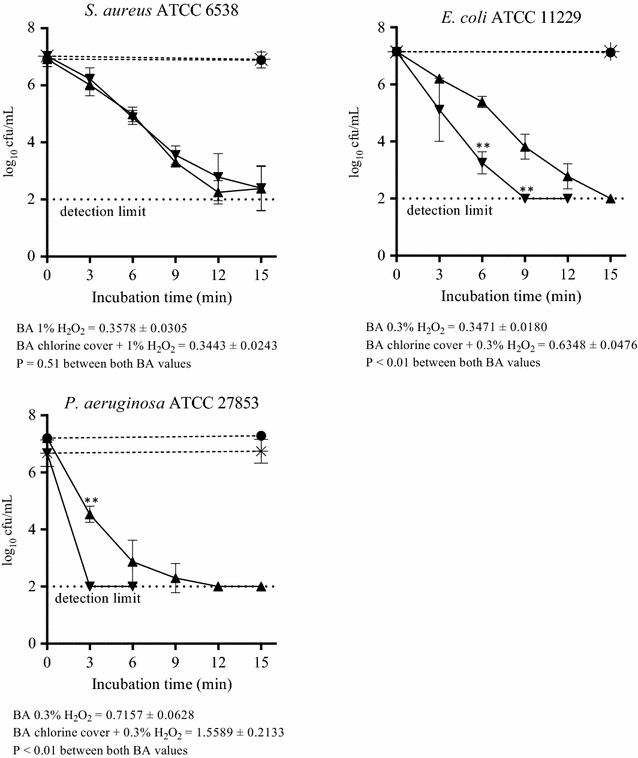
Bactericidal activity of H_2_O_2_ (*filled triangle*) and of H_2_O_2_ against chlorine-covered bacteria that were pretreated with 1% NCT for 1 min (*inverted triangle*). 1% H_2_O_2_ against *S. aureus* ATCC 6538, 0.3% H_2_O_2_ against *E. coli* and *P. aeruginosa*. Controls in phosphate buffer without additives, without (*filled circle*) or with chlorine cover (*Asterisk*). Mean values ± SD of three to four independent experiments. **P < 0.01 versus all other values. BA values calculated as in Fig. 4

When NCT-pretreated, chlorine-covered bacteria were incubated in H_2_O_2_, a differential result was gained, depending on the species used. With *S. aureus*, the chlorine cover did not enhance the susceptibility to H_2_O_2_ (Fig. 8). By contrast, *E. coli* and *P. aeruginosa* were killed significantly more rapidly by H_2_O_2_ if pretreated with NCT (Fig. 8) (*P* < 0.01).

## Discussion

**Scheme 1:**
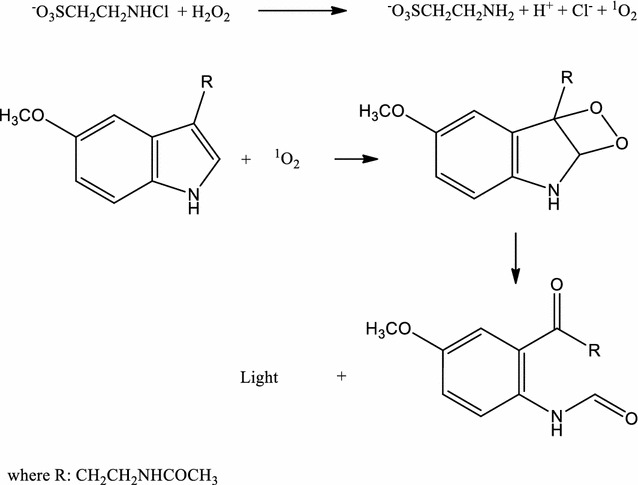
Production of singlet oxygen by NCT and H_2_O_2_ and its reaction with melatonin leading to light emission

Both hydrogen peroxide and NCT are important low reactive components of the oxidative armament of human granulocytes and monocytes and can be used as endogenous antiseptics in human medicine (Baldry 1983; Gottardi et al. 2013; Gottardi and Nagl 2010; Winterbourn and Kettle 2013). For both regards, investigations of the interaction between these molecules may be of interest and contribute to elucidate biological processes. A slow and long-lasting production of singlet oxygen by NCT plus H_2_O_2_ was characterized for the first time in this study. Concentrations of the oxidants were adjusted to reveal good quantitative results. Two procedures were used to monitor the reactions and to highlight the differences between NCT and its precursor HOCl. In the first one, the higher reactivity of HOCl compared to NCT was clearly demonstrated by its rate of consumption. However, the concomitant production of singlet oxygen, monitored by its phosphorescence emission, is uneasy to follow due to the low efficiency of light emission. This was the reason to add melatonin in the medium, since its reactivity with singlet oxygen is well-known and characterized by the efficient emission of chemiluminescence (Lu et al. 2002). Scheme 1 depicts a mechanistic proposal for production of singlet oxygen and the emission of chemiluminescence through generation of a dioxetane intermediate. It is worth of note that there are other oxidative pathways by which melatonin could generate light emission, however, this was not the case using NCT or H_2_O_2_ alone. In addition, the amplification of the light emission provoked by deuterium oxide is an additional evidence of the intermediate singlet oxygen.

We hypothesized that production of ^1^O_2_ would increase the microbicidal activity of NCT and H_2_O_2_ if they were used in combination. For these investigations, we applied higher, clinically applied concentrations and gained reasonable killing times of microorganisms. Actually, against bacteria an additive effect of both compounds was found. It was not strong, but reached high significance compared to the single components. This was true for single incubation time points as well as for the whole killing curves condensed by the integral method (Gottardi et al. 2015), which proved to be of high advantage for comparison of curves. The additive effect was independent of the pH and still present at pH 4. In acidic environment, both single NCT and H_2_O_2_ showed stronger killing than at pH 7, but this was much more pronounced with NCT. Therefore, NCT exerted a stronger bactericidal effect than H_2_O_2_ at pH 4. Active chlorine compounds typically and markedly increase their microbicidal activity at acidic pH due to formation of further oxidizing species and due to loss of negative charges of the bacterial surface [for details see (Gottardi et al. 2013)].

We further hypothesized that the additive effect of NCT and H_2_O_2_ would disappear in the presence of scavengers of oxygen radicals (DMSO, sodium azide). However, this assumption turned out as not true. The combination was still stronger bactericidal, which was further confirmed in the presence of 5% peptone, a general scavenger of oxidants. These results indicate that formed singlet oxygen might not be responsible for this effect. A possible explanation is a combined attack of NCT and H_2_O_2_ on the bacterial cell wall and cell membrane. This may lead to earlier penetration into the microorganism followed by its rapid irreversible inactivation. In this regard, however, we cannot completely discard the involvement of singlet oxygen, since it is possible that azide (N_3_^−^), a charged molecule, and the other scavengers had not total access to the site of generation of singlet oxygen in the cell covers of the bacteria.

Sequential application of NCT and H_2_O_2_, revealing an additive killing effect in Gram-negative bacteria but not in *S. aureus*, rather confirms that singlet oxygen is not responsible. Combined attack and more rapid destruction of the bacterial covers followed by slightly more rapid penetration of oxidation capacity into the bacteria appears to be a conclusive hypothesis in our opinion. Detailed contribution of the multitude of single reaction partners/products are unknown, although the basic chemical reactions have been elucidated (Gottardi et al. 2013; Peskin et al. 2009). From previous studies it is known that the chlorine cover attached by sublethal treatment with NCT does not kill the microorganisms but removes their virulence and causes a lag of regrowth and postantibiotic effect (Gottardi and Nagl 2005; Lackner et al. 2015; Nagl et al. 1999). Therefore, we think that the damage of the surface by the chlorine cover promotes the attack by H_2_O_2_ at least in Gram-negatives. The thicker Gram-positive cell wall seems to resist few min longer to penetration of the used oxidants than the Gram-negative one.

The relatively slow killing of bacteria and fungi by millimolar NCT appears to approximately correlate with its penetration into the cytosol of these organisms. Slow penetration of 1 mM NCT into endothelial cells has been found (Peskin et al. 2005), which was confirmed in our laboratory with 55 mM NCT using keratinocytes (A431), lung epithelial cells (A549), and *Aspergillus fumigatus* (M. Nagl, A. Windisch, unpublished results).

Against fungi, no additive effect was found, at least at the tested concentration. The activity of NCT against fungi is markedly lower than that of H_2_O_2_ in phosphate buffer, probably due to slow penetration (Nagl et al. 2001, 2002). Obviously, the combined attack is not sufficiently strong to kill fungi more rapidly.

Under protein load, H_2_O_2_ showed the expected decrease of activity, while NCT was markedly increased. The latter can be explained by transchlorination from NCT to amino groups of peptone, whereby among others low molecular weight chloramines are formed in equilibrium, which have stronger microbicidal activity (Gottardi et al. 2014). This is particularly true for monochloramine (NH_2_Cl) because of its higher lipophilicity (Gottardi et al. 2007). The enhancement of activity under protein load renders NCT a particularly interesting antiseptic for treatment of infections with high amounts of exudate (Gottardi et al. 2014; Gottardi and Nagl 2013). Moreover, NCT kills fungi much more rapidly in the presence of organic matter than in buffer solution (Gruber et al. 2017; Lackner et al. 2015; Nagl et al. 2001).

Regarding the human defence system, the results of this study may provide evidence that single oxidants cooperate in their attack on invading microorganisms. Despite a high number of studies on highly reactive oxidants such as hypochlorite and long-lived ones such as chloramines, this aspect appears to have been underestimated up to date. Hydrogen peroxide and NCT are moderately enhanced in their bactericidal activity if applied in combination. The relatively slowly produced singlet oxygen seems not to be responsible for this effect. Consequences are not fully foreseeable presently and may comprise the understanding of the human defence system and application of oxidants as antiseptics.

## Abbreviations

ANOVA: analysis of variance
BA: bactericidal activity
Cfu: colony forming units
DMSO: dimethylsulphoxide
NCT: N-chlorotaurine
ROS: reactive oxygen species
SD: standard deviation
